# Acne Keloidalis Nuchae in a Caucasian Non-Hispanic Woman With Metabolic Syndrome and Autoimmune Thyroiditis: A Case Report

**DOI:** 10.7759/cureus.59119

**Published:** 2024-04-27

**Authors:** Boyana Anatolieva, Veselin Kirov, Silvia Ganeva

**Affiliations:** 1 Dermatology, Medical University, Pleven, BGR; 2 Dermatooncology, University Hospital, Pleven, BGR; 3 Clinic of Endocrinology, Heart and Brain Hospital, Pleven, BGR

**Keywords:** autoimmune hypothyroiditis, insulin resistance, hyperinsulinemia, metabolic syndrome, acne keloidalis nuchae

## Abstract

Acne keloidalis nuchae (AKN) is a rare dermatological condition primarily observed in men of African descent. We present a remarkable case of AKN in a 38-year-old Caucasian non-Hispanic woman with metabolic syndrome and autoimmune thyroiditis. After appropriate treatment during the one-year follow-up (including oral antibiotics, insulin sensitizers, levothyroxine, spironolactone and liraglutide), the patient demonstrated a visible reduction in plaque size and improvement of overall symptoms. Importantly, this improvement persisted even in the absence of topical treatment, further supporting the notion that hormonal abnormalities may play a significant role in the pathogenesis of AKN.

This case report highlights the potential link between AKN and endocrinologic disorders, such as metabolic syndrome and autoimmune thyroiditis. However, further research is warranted to elucidate the underlying mechanisms and establish the causative relationship. Early recognition, appropriate management of associated conditions, and tailored treatment strategies may lead to better outcomes and improved quality of life.

## Introduction

Acne keloidalis nuchae (AKN) is a rare dermatological condition primarily observed in men of African descent with a male to female ratio of up to 20:1. AKN usually begins after puberty and rarely after the age of 50 [1]. Increase in the activity of the sebaceous glands, androgen receptor sensitivity of the hair follicles, and the effect of the increase in hormone levels itself during puberty are proposed explanations [2]. 

AKN begins as folliculitis with formation of perifollicular papules, pustules and crusts typically located on the posterior neck. With time the papules coalesce to form fibrotic plaques, extending beyond the hair follicles, resembling a keloid. Alopecia is observed in the affected areas due to the destruction of hair follicles from the inflammatory process and subsequent fibrosis [3]. The aetiology and pathogenesis of AKN are still unclear, but they are considered multifactorial. Possible inciting factors that have been suggested include trauma, inflammation, infection, hormonal, genetics and ingrown hairs. It is unknown why the predilection site for this condition is the occipital region. Several explanations for this phenomenon have been suggested, but not proven, including elevated levels of androgens in the scalp, increase in mast cells and dermal papillary dilatation in the nuchal area [2] as well as friction of the scalp skin folds and obesity [4]. Most studies describe a history of chronic friction in the area with short haircuts, frequent wearing of tight collars, hats and helmets in certain professions (e.g. athletes), but a direct causative mechanism has not been proven [2,3,5,6].

The role of genetics has been considered due to the fact that AKN occurs mainly in people of African descent who tend to have curly hair that often becomes ingrown and causes chronic inflammation [5]. However, histology of early lesions of AKN does not suggest that ingrown hairs stimulate the inflammation in AKN [6].

It is certain that inflammation plays a role in the pathogenesis of AKN, however, it is not clear whether it is primary or secondary. AKN is classified as a primary form of inflammatory scarring alopecia with no proven inciting agents. The inflammation causes oedema and the formation of papules in early disease stages. With the deposition of collagen in the dermis and fibrosis formation, destruction of sebaceous glands is observed, however, it is unclear whether they are the main target of inflammatory cells or get destroyed in the process [7]. The role of bacteria (mainly *Staphylococcus aureus*) in the pathogenesis of AKN is believed to be secondary as the infection is not found in all cases [3].

There is an increasing number of studies in recent years suggesting a link between AKN and metabolic syndrome, but larger-scale studies are needed to prove this relationship [8-10]. Metabolic syndrome (MetS) is defined as a cluster of risk factors that are associated with insulin resistance and increase the risk for developing cerebrocardiovascular disease and type 2 diabetes mellitus. These risk factors consist of obesity and hyperlipidaemia, hyperinsulinaemia, hypertension, and hyperglycaemia [11]. Two well-known skin markers for MetS and obesity in particular are acanthosis nigricans and acrochordons, both of which can be attributed to activation of insulin-like growth factor 1 (IGF-1) receptors in the setting of hyperinsulinaemia [12]. 

Hypothyroidism disorders are characterized by lower production of thyroid hormones. Autoimmune thyroiditis, also known as Hashimoto thyroiditis, is an autoimmune disease that destroys thyroid cells by cell and antibody-mediated immune processes [13]. A study by Valdman-Grinshpoun et al. demonstrated an increased risk of hypothyroidism among female patients with AKN [14]. The higher prevalence of females among individuals with AKN and thyroid diseases corresponds with the typical female predominance observed in thyroid diseases, particularly autoimmune thyroid disease. It has been hypothesized that hypothyroidism may be associated with acanthosis nigricans, but it has not yet been proven in large-scale studies [15].

Treatment of AKN is usually challenging and involves a combination of topical, systemic and surgical modalities, including oral and topical antibiotics, topical and intralesional corticosteroids, surgery and lasers. Treatment requires months and recurrences are common. Thus the primary focus should be prevention when possible. More large-scale studies are needed to determine optimal treatment plans [16].

## Case presentation

**Figure 1:**
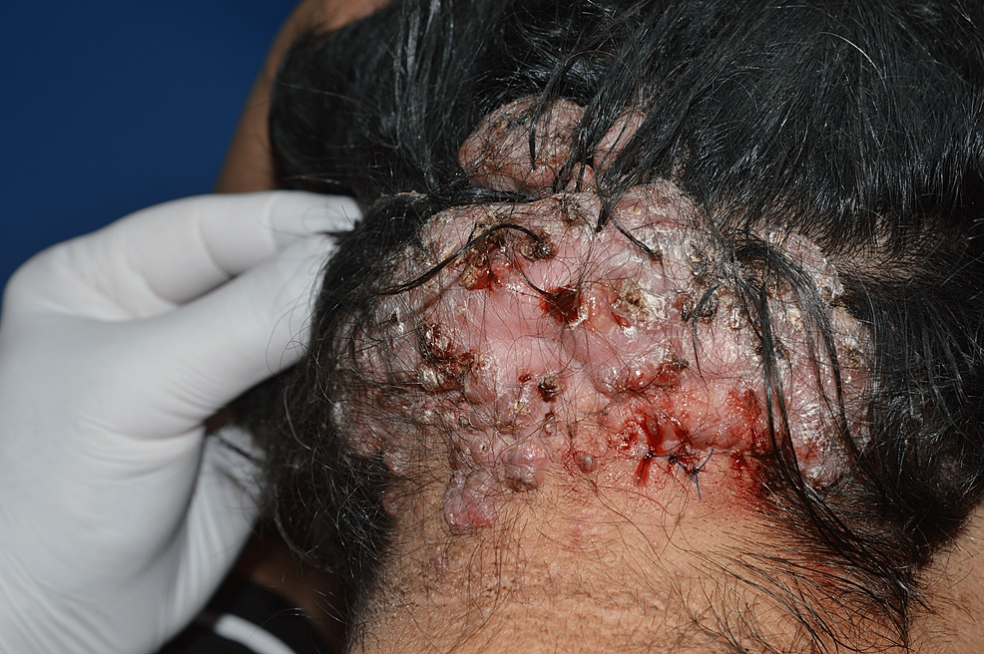
Keloid-like fibrous plaque measuring at 15/8 cm located at the nape of the neck and occipital region. An uneven surface is seen, covered with multiple perifollicular papules, pustules and crusts. Polytrichia can be seen, characterized by clusters of hairs coming out of a single hair follicle.

We present a remarkable case of AKN in a 38-year-old Caucasian non-Hispanic woman with metabolic syndrome and autoimmune thyroiditis. The patient presented with a two-year history of persistent, pruritic firm bumps on the posterior neck and occipital region of the scalp which began developing after a haircut. Physical examination revealed firm, raised, keloid-like lesions consistent with AKN (Figure 1).

**Figure 2:**
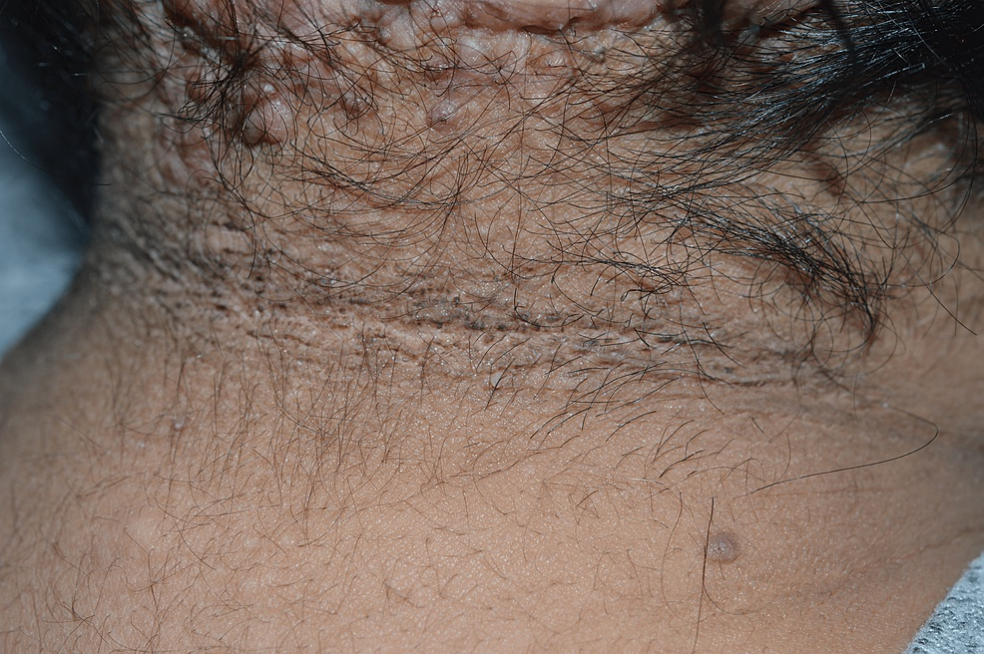
Acanthosis nigricans on the back of the neck.

**Figure 3:**
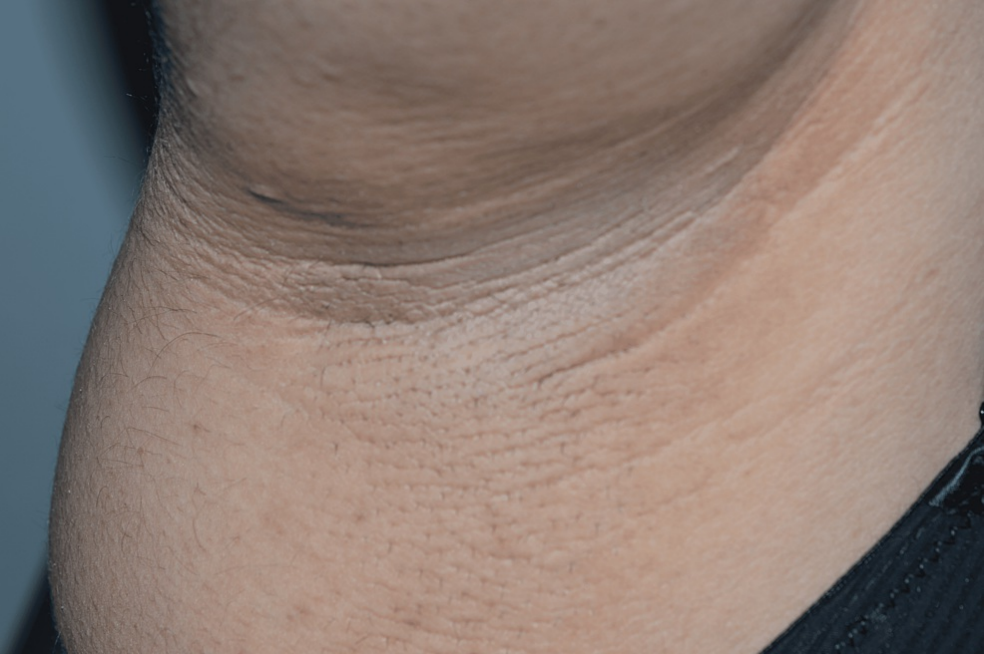
Acanthosis nigricans in the axillary region.

**Figure 4:**
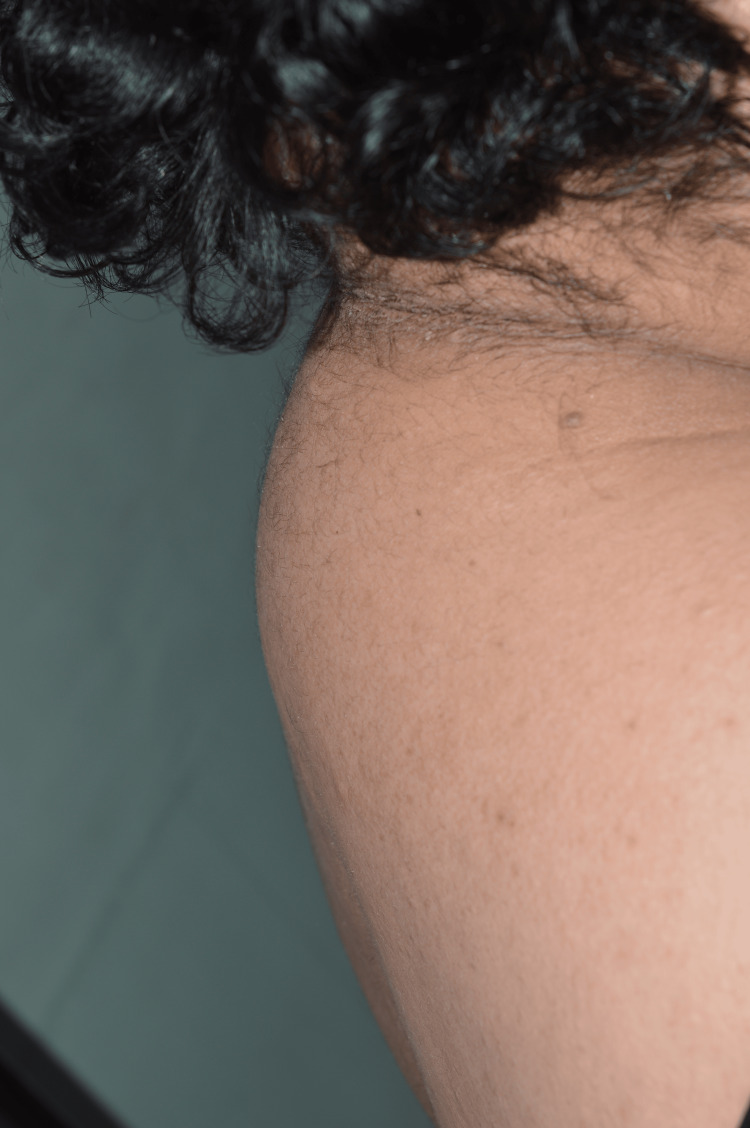
Buffalo hump on C7.

Further clinical inspection revealed brown velvet-like plaques of pseudoacanthosis nigricans around the neck (Figure 2) and in both axillae (Figure 3) as well as fibroepithelial polyps (acrochordons) in the same areas, suggestive of insulin resistance. The patient also had acne lesions on the face and upper back and signs of hirsutism on the face, abdomen and inner thighs, suggestive of hyperandrogenism. Additional findings were a “buffalo hump” or deposition of fat at the back of the neck (Figure 4) as well as swelling of the feet.

During the checkup the patient appeared bradypsychic and lethargic, with minimal facial expressions while talking. She had a medical history of hypothyroidism and admitted not cooperating with her treatment in the last few years. She had no other complaints and no family history of any diseases. 

A swab was taken from a pustule on the keloid-like plaque and sent for microbiology testing. Staphylococcus aureus was isolated from the culture and appropriate antibiotic treatment was administered according to the antibiogram - oral azithromycin three times a week for four weeks (standard acne vulgaris regimen in Bulgaria).

**Figure 5:**
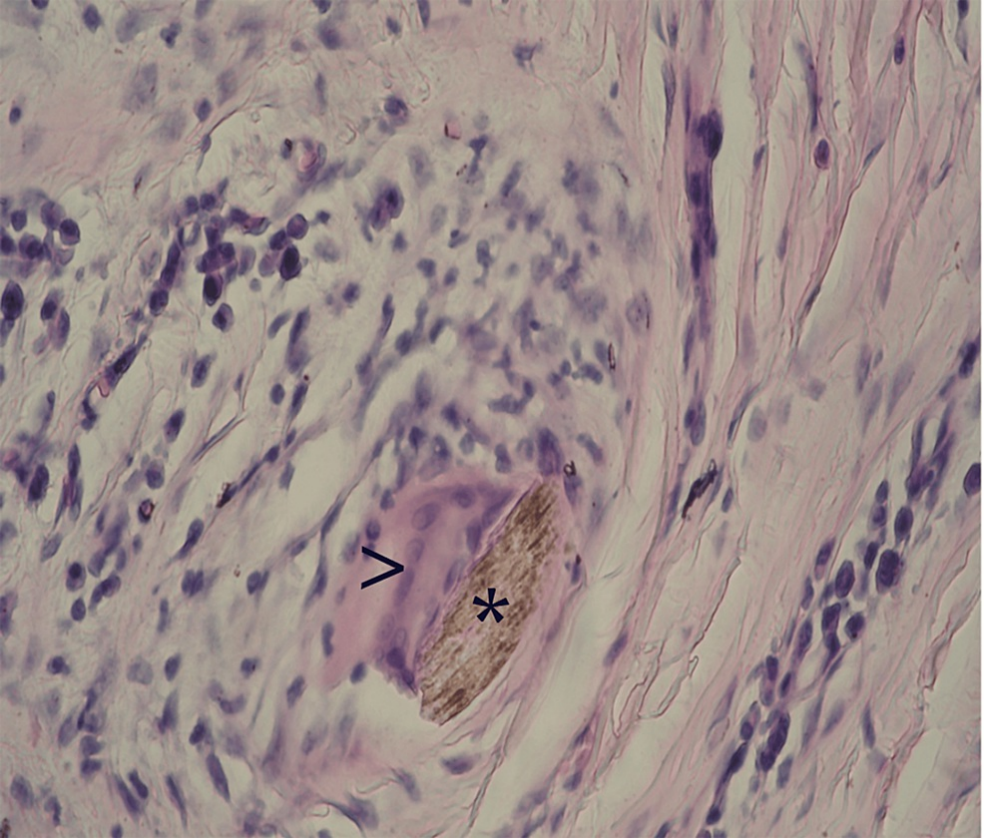
400x HE. Mixed inflammatory infiltrate with a foreign body giant cell (>) around a hair shaft (*).

**Figure 6:**
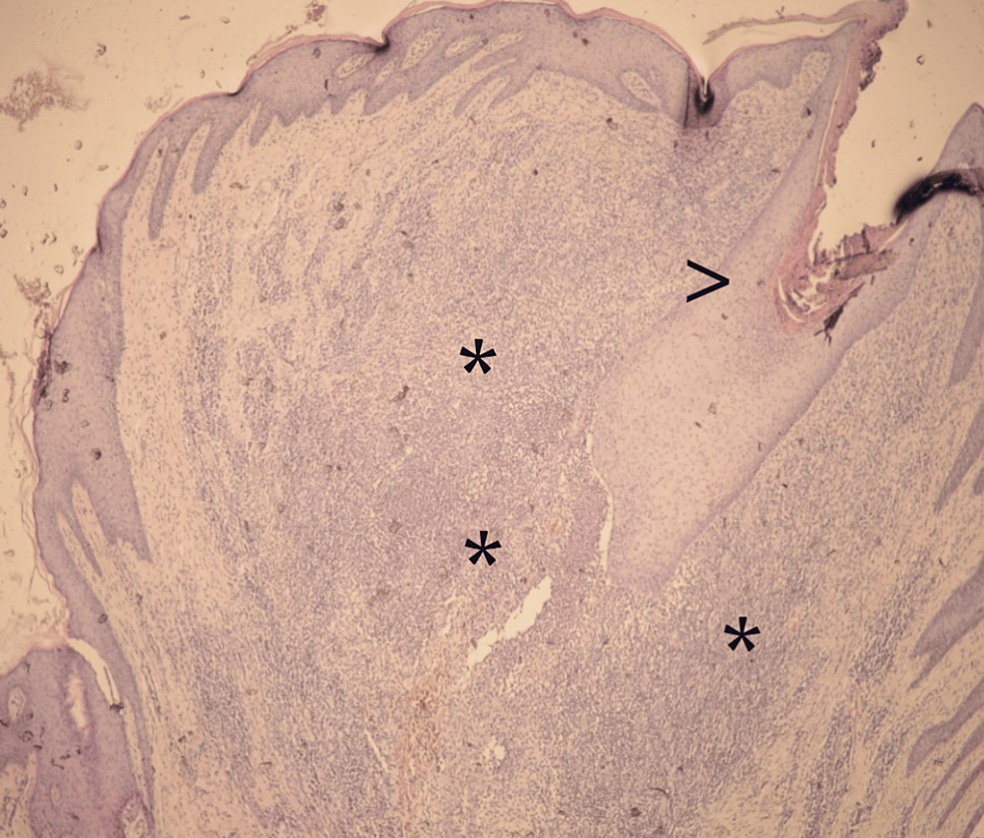
100x HE. Hair follicle (>) surrounded by a mixed chronic inflammatory infiltrate (*), dermal fibrosis and destruction of adnexal structures (absence of follicle-associated sebaceous glands).

An excisional skin biopsy was taken from the plaque. The pathology report found hair follicles surrounded by a mixed chronic inflammatory infiltrate with foreign body giant cells (Figure 5), fibrosis of the dermis and destruction of adnexal structures (Figure 6). Keratin plugs were observed in the openings of the hair follicles. The findings were consistent with acne keloidalis nuchae.

Following the dermatologic evaluation, the patient was admitted to the Clinic of Endocrinology for comprehensive testing and diagnosis. On admission, physical examination revealed elevated arterial blood pressure (180/100 mmHg) and visceral obesity with a body mass index (BMI) of 38 kg/m2.

An oral glucose tolerance test (OGTT) was performed to assess glucose metabolism. The blood glucose curve demonstrated a normal response but the insulin levels (both the fasting and stimulated) were found to be extremely elevated. The calculated homeostasis model assessment-estimated insulin resistance (HOMA-IR) index, a marker of insulin resistance, was notably high at 80.66, correlating with extreme insulin resistance (reference range <2.5) (Table 1).

**Table 1:** Oral glucose tolerance test *IRI - Immunoreactive insulin. The base insulin reference range is <16. **Homeostasis model assessment-estimated insulin resistance (HOMA-IR) - the HOMA index is calculated by the equation Blood sugar x base IRI / 22.5. Reference range - <2.5 - no insulin resistance (IR); 2.5 - 5 - borderline; >5 - severe IR.

Time at testing	Blood glucose (mmol/L)	IRI*	HOMA-IR**
0 min	5.2	349	80.66
60 min	8.02	528	
120 min	7.42	176	

Thyroid function tests revealed elevated levels of thyroid-stimulating hormone (TSH) along with elevated titers of antithyroid antibodies. The free thyroxine (fT4) levels were found to be low, suggestive of uncompensated hypothyroidism in the context of Hashimoto's thyroiditis (Table 2). 

**Table 2:** Additional laboratory findings TSH: thyroid-stimulating hormone, fT4: free thyroxine, TPO: thyroid peroxidase, LH: luteinizing hormone, FSH: follicle-stimulating hormone

Indicator	Value	Reference range
TSH	71.6	0.2 - 4.0 mIU/l
fT4	2.8	7.0 - 18.0 pmol/l
TPO	734.0	<70
Prolactin	11.6	<25 ng/mL
LH	6.5	0.5 - 7.6 mIU/l
FSH	11.4	1.2 - 13.4 mIU/l
Testosterone	2.6	<2.7 pmol/l
Cholesterol	6.4	<5.2 mmol/l
Uric acid	470	<340 mcmol/l

The ultrasound examination of the thyroid gland showed focal and diffuse structural changes consistent with autoimmune thyroiditis. Based on the clinical, laboratory and ultrasound findings the patient was diagnosed with uncompensated and untreated hypothyroidism and chronic autoimmune thyroiditis of Hashimoto. 

The patient's laboratory results also revealed elevated levels of cholesterol and uric acid (Table 2) and these findings, combined with arterial hypertension, visceral obesity, hyperinsulinaemia and insulin resistance, supported a diagnosis of metabolic syndrome.

Although the patient exhibited clinical signs of hyperandrogenaemia, such as hirsutism and acne, her total testosterone levels in sera were within the reference range, but sex hormone-binding globulin (SHBG) and free testosterone were not investigated and did not support the observed manifestations. It was recommended that the patient undergo testing for SHBG, dehydroepiandrosterone sulfate (DHEAS) and androstenedione to further investigate the underlying hormonal imbalances. Unfortunately, the patient did not follow up.

Upon further questioning the patient shared she had been experiencing irregular menstrual cycles since her menarche at 16 years of age and so she was consulted with an OB/GYN specialist. Although no ultrasound signs of polycystic ovary syndrome (PCOS) were observed, additional testing to evaluate adrenal and pituitary gland function was recommended. However, the patient did not follow up with these recommendations.

A treatment plan was initiated consisting of substitutive levothyroxine 125 mcg/d, metformin 2000 mg/d as an insulin sensitizer, liraglutide 0.6 mg per day subcutaneous as a glucagon-like peptide 1 (GLP-1) receptor agonist, spironolactone 50 mg/d as a pluripotent androgen blocker.

**Figure 7:**
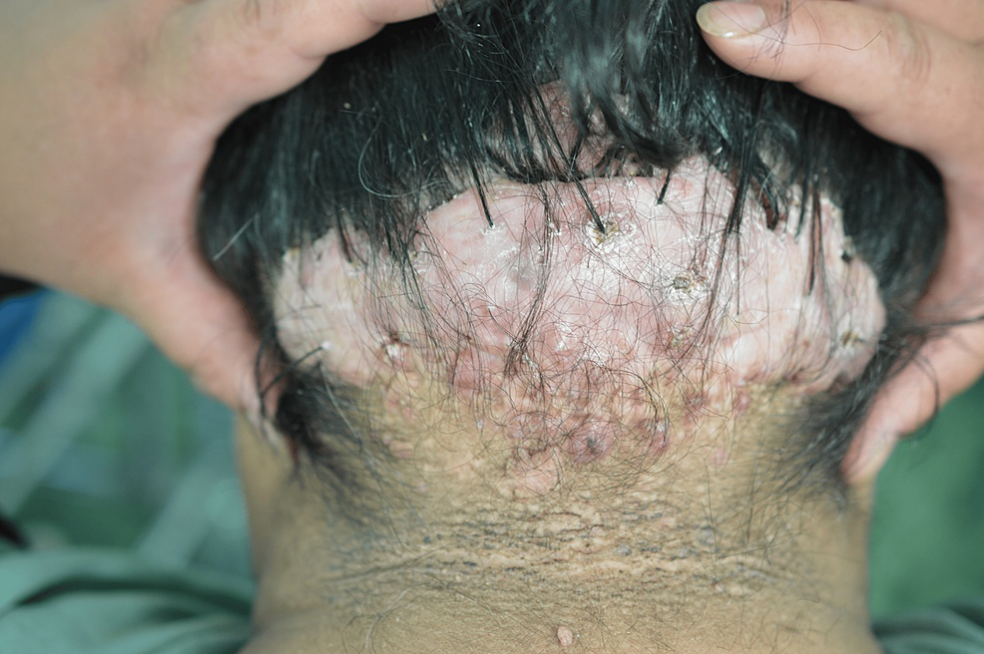
Patient at follow-up after one year of treatment. Plaque is reduced in size and no pustules are seen.

The patient returned for a follow-up examination after a year of treatment. Despite the absence of follow-up laboratory tests and the patient's confession of poor treatment adherence, significant improvement was observed in her clinical presentation (Figure 7). There was a visible reduction in the fibrotic plaque size and absence of acne lesions on her skin. There was no improvement of the acanthosis nigricans in the nuchal and axillary folds. There was no reduction in the number of acrochordons in these areas but there were no new ones either. The patient reported a reduction in weight and a marked resolution of her bradypsychic state could be observed, indicating an improvement in her overall well-being. 

## Discussion

Despite inconsistent treatment adherence, the control of underlying metabolic abnormalities seemed to contribute to the spontaneous improvement of the fibrous plaque, suggesting a potential pathogenetic link between AKN and metabolic disorders. Importantly, this improvement persisted even in the absence of topical treatment, further supporting this notion.

Histology

The histological features described in AKN include folliculocentric inflammation (folliculitis and perifolliculitis), dilatation of the follicular infundibulum with follicular keratin plugs, follicular and perifollicular fibrosis and destruction of adnexal structures in the form of absence of sebaceous glands [7,17].

AKN and hyperinsulinaemia

Most literature cites mechanical trauma and chronic skin inflammation of the neck as the main factor for development of AKN, and subsequently metabolic disorders are discovered. In recent years more and more reports are emerging suggesting a pathogenetic link between AKN lesions and metabolic abnormalities [8-10].

In the setting of hyperinsulinaemia and insulin resistance, there is an activation of insulin receptors as well as IGF-1 receptors with the appearance of hybrid IGF-1 receptors [12]. All of these receptors can be found on the cell surface of keratinocytes and fibroblasts. Insulin receptors and IGF-1 receptors are structurally similar, and their activation stimulates mitogen-activated protein (MAP) kinase activity, which regulates cellular processes such as growth, proliferation, gene expression, and apoptosis suppression. This molecular pathway could provide an explanation for the observed clinical manifestations, including acanthosis nigricans, growth of acrochordons, formation of follicular keratin plugs, and increased sebaceous gland activity leading to more severe acne in these patients [18]. 

AKN and thyroiditis

According to a study by Valdman-Grinshpoun et al., the risk of developing hypothyroidism was 1.85 times higher among patients with AKN compared to the control group. Furthermore, patients with concomitant AKN and thyroid disease were significantly older at the onset of AKN, had a more marked female predominance, and had a higher burden of comorbidity. The greater dominance of females among patients with AKN and thyroid diseases aligns with the general female preponderance in thyroid diseases and autoimmune thyroid disease, in particular. No association between AKN and hyperthyroidism was found [14].

Acanthosis nigricans and acrochordons

The reduction of the size of the fibrotic plaque and clearance of acne lesions in this patient can possibly be attributed to the reduction of insulin levels and antibiotic therapy.

There was no improvement observed in the acanthosis nigricans and no reduction in the number of acrochordons. Notably, there was no increase in the number of acrochordons which supports the hypothesis that the pathology of AKN may be linked to the IGF-1/MAPK axis [18]. Further research is needed to confirm this observation.

## Conclusions

In conclusion, this case report adds to the accumulating evidence suggesting an association between metabolic abnormalities, autoimmune thyroiditis, and AKN in a Caucasian woman. Clinicians should be vigilant for the presence of endocrinologic comorbidities in patients with AKN, even in those outside the typical demographic profile. 

The findings of this case report raise important questions regarding the pathogenesis of AKN and its relationship with metabolic disorders. The activation of insulin and IGF-1 receptors, along with their subsequent effects on cell growth and proliferation, may contribute to the cutaneous findings in this patient. This case underscores the importance of a multidisciplinary approach and further research to better understand the complex pathogenetic mechanisms and establish a causative relationship between AKN and metabolic disorders.
